# Heterogeneity and prognostic significance of mast cell subsets in the tumor microenvironment of prostate cancer

**DOI:** 10.1007/s00262-026-04363-6

**Published:** 2026-03-24

**Authors:** Ruizhe Fang, Shuyang Zhao, Minggui Si, Yinshi Zhang, Ruicong Xu, Jingjia Li

**Affiliations:** 1 Department of Urology, The Fourth Affiliated Hospital of Harbin Medical University, Harbin, 150001 China; 2 College of Pharmacy, Harbin Medical University, Harbin, 150081 China; 3 Department of Urology, Jilin Hospital of Beijing Anzhen Affiliated Hospital of Capital Medical University, Changchun, 130022 China

**Keywords:** Prostate cancer, Tumor microenvironment, Mast cells, Prognosis, Marker genes

## Abstract

**Background:**

Prostate cancer (PC) is one of the malignant tumors with high incidence and mortality worldwide in men. Immune cells in the tumor microenvironment (TME), particularly mast cells, play important roles in tumor progression and prognosis. However, the dual roles of mast cells in PC have not been fully elucidated.

**Methods:**

In this study, single-cell RNA sequencing (scRNA-seq), bulk RNA sequencing, and bioinformatics analyses were performed to investigate the heterogeneity of mast cell subsets in the TME of PC and their association with prognosis. Single-cell data from 39 PC tumor samples were analyzed, and prognostic prediction was validated using datasets including HMU, TCGA, and MSKCC cohorts. Non-negative matrix factorization was applied to cluster mast cell subsets, followed by analyses of subset-specific gene markers, transcription factor activity, and biological pathways. Survival analysis and ROC curve evaluation were conducted to assess prognostic value.

**Results:**

Mast cells in the TME of PC were classified into two distinct subsets, each characterized by unique gene markers and functional pathways. Mast cell1 was highly associated with pro-tumorigenic pathways, whereas mast cell2 predominantly exhibited antitumor immune regulatory properties. High expression of mast cell1 signatures was correlated with poorer survival outcomes, while high expression of mast cell2 signatures was associated with better survival. Key marker genes such as BIRC3 and FOS were identified as potential prognostic factors, high BIRC3 expression was significantly associated with unfavorable prognosis, whereas high FOS expression correlated with favorable prognosis.

**Conclusion:**

This study revealed functional heterogeneity of mast cell subsets in the TME of PC and their distinct roles in tumor progression. The identification of subset-specific marker genes provides novel molecular targets for clinical diagnosis, prognostic prediction, and personalized therapy in PC.

**Supplementary Information:**

The online version contains supplementary material available at 10.1007/s00262-026-04363-6.

## Introduction

Prostate cancer (PC) is the second most commonly diagnosed malignancy among men worldwide [1]. According to reports from the World Health Organization, it is also the second leading cause of cancer-related mortality in the global male population [2]. With the ongoing trend of population aging, the number of new PC cases is projected to reach 2.9 million by 2040 [3]. Although notable advances have been made in recent years in early screening, surgical management, chemotherapy, targeted therapies, and immunotherapy, inevitable treatment failure and subsequent disease progression remain pressing clinical challenges [4]. Therefore, PC continues to pose a formidable therapeutic burden, highlighting the urgent need for deeper investigations into its underlying mechanisms and for the development of more effective treatment strategies. The initiation and progression of PC involve complex multifactorial interactions, including genetic susceptibility, environmental influences, and alterations in the tumor microenvironment (TME) [5]. The TME refers to a highly intricate milieu composed of tumor cells and surrounding non-malignant components, such as stromal cells, vasculature, immune cells, extracellular matrix, and signaling molecules [6]. The TME plays a pivotal role in cancer initiation, progression, metastasis, and therapeutic responses [7]. Among immune cell populations, mast cells have been increasingly recognized as key contributors within the TME.

Mast cells are bone marrow-derived immune cells that are widely distributed throughout various tissues in the body, particularly in the skin, lungs, gastrointestinal tract, and urinary tract [8, 9]. They play essential roles in immune responses primarily through the release of a broad spectrum of bioactive mediators stored in intracellular granules, including histamine, cytokines, chemokines, proteases, and lipid-derived mediators. These mediators regulate diverse physiological processes such as immune modulation, inflammation, and tissue repair [10]. The role of mast cells in the TME has long been a major focus of research. In addition to their well-established involvement in allergic reactions, antimicrobial immunity, and tissue remodeling, mast cells have also been increasingly recognized as being closely associated with tumor progression and prognosis [11]. By secreting a variety of mediators, mast cells can modulate immune responses within the TME, thereby influencing tumor growth, metastasis, and therapeutic outcomes [12].

The role of mast cells in cancer is complex and bidirectional. Accumulating evidence indicates that mast cell abundance is closely associated with cancer prognosis [13]. Several studies have reported that mast cell infiltration within tumor tissues correlates with tumor aggressiveness, metastatic potential, and therapeutic resistance, suggesting that mast cells may facilitate tumor progression through the secretion of pro-tumorigenic factors [14, 15]. Conversely, other studies have demonstrated that mast cells may exert antitumor effects to some extent by enhancing antitumor immune responses and inducing apoptosis in tumor cells. Within the TME of cancer, mast cells represent a critical immune cell population with substantial immunoregulatory capacity. Through the release of bioactive mediators, mast cells interact with tumor-associated immune cells, such as T cells, B cells, and macrophages, to shape local immune responses [16]. Moreover, mast cells can recruit additional immune cells to the tumor site by releasing cytokines and chemokines, thereby influencing immune surveillance and regulating tumor immune evasion.

The development of PC is intimately linked to complex cellular crosstalk within the TME. Although mast cells are recognized as critical immunoregulatory components, their roles in PC remain poorly defined and sometimes contradictory. Given their potential to shape immune surveillance, promote tumor dissemination, and influence responsiveness to immunotherapy, it is urgently necessary to clarify the functional and mechanistic significance of mast cells in PC progression. Therefore, the present study was designed to elucidate the role of mast cells and the molecular pathways through which they modulate the TME of PC, thereby providing potential therapeutic targets and a rationale for the development of more effective therapeutic strategies.

## Materials and methods

### Patients

This study included 106 formalin-fixed, paraffin-embedded (FFPE) specimens of PC collected at the Fourth Affiliated Hospital of Harbin Medical University between 2010 and 2020 (Table 1 and Supplementary Table 1). Eligible patients met the following inclusion criteria: (1) age between 18 and 75 years; (2) histopathological confirmation of prostate adenocarcinoma based on postoperative specimens; (3) completion of R0 surgical resection; and (4) availability of at least 5 years of follow-up data. Patients were excluded if they (1) received neoadjuvant therapy prior to surgery or (2) lacked essential clinicopathological information and/or complete postoperative follow-up and treatment records.

**Table 1:** Prostate cancer-related datasets

Number	Samples	Platform	Type
HMU cohort	106	Illumina	Bulk RNA-seq
MSKCC cohort	150	Illumina	Bulk RNA-seq
TCGA cohort	497	Illumina	Bulk RNA-seq
GSE181294	39	Illumina	scRNA-seq

### RNA isolation and sequencing

Total RNA quality and quantity were evaluated through a standardized workflow. First, RNA degradation and contamination were assessed by 1% agarose gel electrophoresis. RNA purity and concentration were then measured using a NanoPhotometer® spectrophotometer, and RNA integrity was further examined with the RNA Nano 6000 Assay Kit on the Bioanalyzer 2100 system [17]. Only qualified RNA samples were subjected to library construction. For library preparation, mRNA was enriched using oligo(dT)-conjugated capture beads, followed by purification with binding and washing buffers. Purified mRNA was randomly fragmented into approximately 100–200 nt and subsequently reverse-transcribed into cDNA. The resulting cDNA was purified using DNA Clean Beads, after which adapters containing unique molecular identifiers (UMIs) were ligated. Libraries were then amplified by PCR, purified again, and eluted in nuclease-free water. After library construction, samples were pooled and sequenced on an Illumina NovaSeq 6000 platform, generating approximately 6 Gb of raw data per sample with 150-bp paired-end reads [18].

Raw sequencing reads in FASTQ format were initially processed using in-house Perl scripts. During preprocessing, reads containing adapter sequences, poly-N stretches, or low-quality bases were filtered out to obtain high-quality clean reads, which were used in all downstream analyses. The reference genome index (Homo sapiens GRCh38.103) was built using STAR (v2.7.11b), and clean reads were aligned to the reference genome using STAR. Gene-level expression counts were quantified with RSEM (v1.3.3) [19].

### Acquisition and preprocessing of single-cell data

The single-cell RNA sequencing (scRNA-seq) dataset of PC was obtained from the publicly available data released by Hirz et al. and comprised a total of 171,510 cells from 39 tumor tissue samples (Table 1) [20]. The scRNA-seq data were processed and converted into a Seurat object using the R package Seurat. Quality control was performed using three criteria: (i) Genes expressed in fewer than 5 cells were excluded; (ii) cells expressing fewer than 100 genes were removed; and (iii) cells with > 5% mitochondrial gene expression were filtered out. Subsequently, the data were normalized using the NormalizeData function, and the top 2000 highly variable genes were identified using FindVariableFeatures. Principal component analysis (PCA) was performed with RunPCA, and the top 20 principal components were selected for downstream cell clustering. Differentially expressed genes (DEGs) for each cluster were identified using FindAllMarkers. Genes were considered significantly differentially expressed when meeting the criteria of FDR < 0.05 and log2(fold change)∣ > 0.25.

Major cell types identified in the dataset were annotated based on well-established canonical marker genes, including CD3D, CD8A, CD4, CD56, FOXP3, CD79A, MS4A1, CD14, CD68, COL1A2, COL3A1, VWF, PECAM1, EPCAM, TPSAB1, and CPA3. These markers were used to classify cells into major populations such as T cells, NK cells, regulatory T cells (Tregs), B cells, myeloid cells, fibroblasts, endothelial cells, epithelial cells, and mast cells, among others.

### Acquisition and preprocessing of bulk transcriptomic data

Bulk transcriptomic data for PC were comprehensively retrieved from public databases, including GEO, cBioPortal (MSKCC), and TCGA. A total of four independent cohorts were included in this study, comprising 792 tumor samples in total (Table 1).

### Cell-cell interaction analysis

We employed the Python package CellPhoneDB (v2.0) to construct a cell-cell communication network within the tumor immune microenvironment, primarily following the default settings of the software [21]. Putative interaction strength between two cell subsets was inferred based on the expression levels of ligand–receptor pairs. Subsequently, interactions were filtered according to statistical significance (*P* < 0.05) to remove non-significant links. Specifically, when a given cell population expressed a receptor or ligand, the corresponding interaction was defined as an incoming (input) or outgoing (output) signal, respectively. Biologically relevant ligand–receptor pairs were examined across different cell subsets. The relative expression levels of ligand–receptor pairs (*z*-scores) and adjusted *P* values were visualized using dot plots or interaction heatmaps.

### Prediction of transcription factor activity using SCENIC

The SCENIC Python workflow (v0.12.1) was applied to infer gene regulatory networks and evaluate transcription factor (TF) activity using default parameters [22]. The normalized expression matrix of target cells served as the input. During the analysis, transcription factor binding motif databases for Homo sapiens from RcisTarget and GRNBoost were utilized, which were retrieved from the official SCENIC resources. Regulon-specificity scores were generated to quantify TF activity specificity, and active TFs were identified based on a binarized regulon activity matrix. In addition, differential TF activity was assessed from the AUC matrix using the Wilcoxon rank-sum test, with thresholds set at FDR < 0.05 and Fold change > 2. The final results were visualized using heatmaps.

### CytoTRACE analysis

The CytoTRACE algorithm, developed by Gulati et al. is an advanced computational approach for analyzing scRNA-seq data [23]. It captures, refines, and quantifies gene expression patterns that are highly correlated with single-cell gene counts, thereby enabling robust inference of cellular developmental potential. After CytoTRACE computation, each cell is assigned a score representing its stemness state within the dataset. CytoTRACE has been extensively validated in large-scale datasets and has been shown to outperform conventional stemness assessment methods in predicting differentiation status from scRNA-seq data. In this study, we applied the R package CytoTRACE (v0.3.3) to calculate CytoTRACE scores for malignant cells, with values ranging from 0 to 1. Higher scores indicate stronger stemness (i.e., a lower degree of differentiation), whereas lower scores reflect reduced stemness (i.e., a higher degree of differentiation).

### Pseudotime trajectory analysis

Monocle2 (v2.20.0) was used to perform pseudotime trajectory analysis to infer differentiation trajectories during cellular development [24]. After extracting the UMI count matrix from the Seurat object, a Monocle object was constructed using the newCellDataSet function. For trajectory inference, genes with a mean expression level greater than 0.1 were selected. Dimensionality reduction was then carried out using the DDRTree algorithm, and cells were ordered along the inferred trajectory using the orderCells function.

### Functional annotation and gene set enrichment analysis (GSEA)

Functional enrichment analyses were performed using the Gene Ontology (GO) database, including the three major GO categories: biological process (BP), cellular component (CC), and molecular function (MF). Significantly enriched GO terms were identified using Fisher’s exact test, and terms with a *q* value ≤ 0.05 were considered statistically significant. In addition, GSEA was conducted to further characterize biological processes and signaling pathways. The results were summarized using the normalized enrichment score (NES), and statistically significant gene sets were visualized based on their NES values.

### Statistical analysis

The optimal cutoff value for stratifying patients into different groups was determined using the surv_cutpoint function implemented in the R package survminer (v0.4.9). Survival outcomes across different patient subtypes were evaluated using Kaplan-Meier survival curves, which were generated with the survminer and survival (v3.3-1) packages. Differences between groups were assessed using a two-sided log-rank test. All statistical analyses were conducted in *R* software (v4.2.0), and a *P* value < 0.05 was considered statistically significant.

## Results

### Single-cell landscape of the tumor microenvironment of prostate cancer

**Fig. 1:**
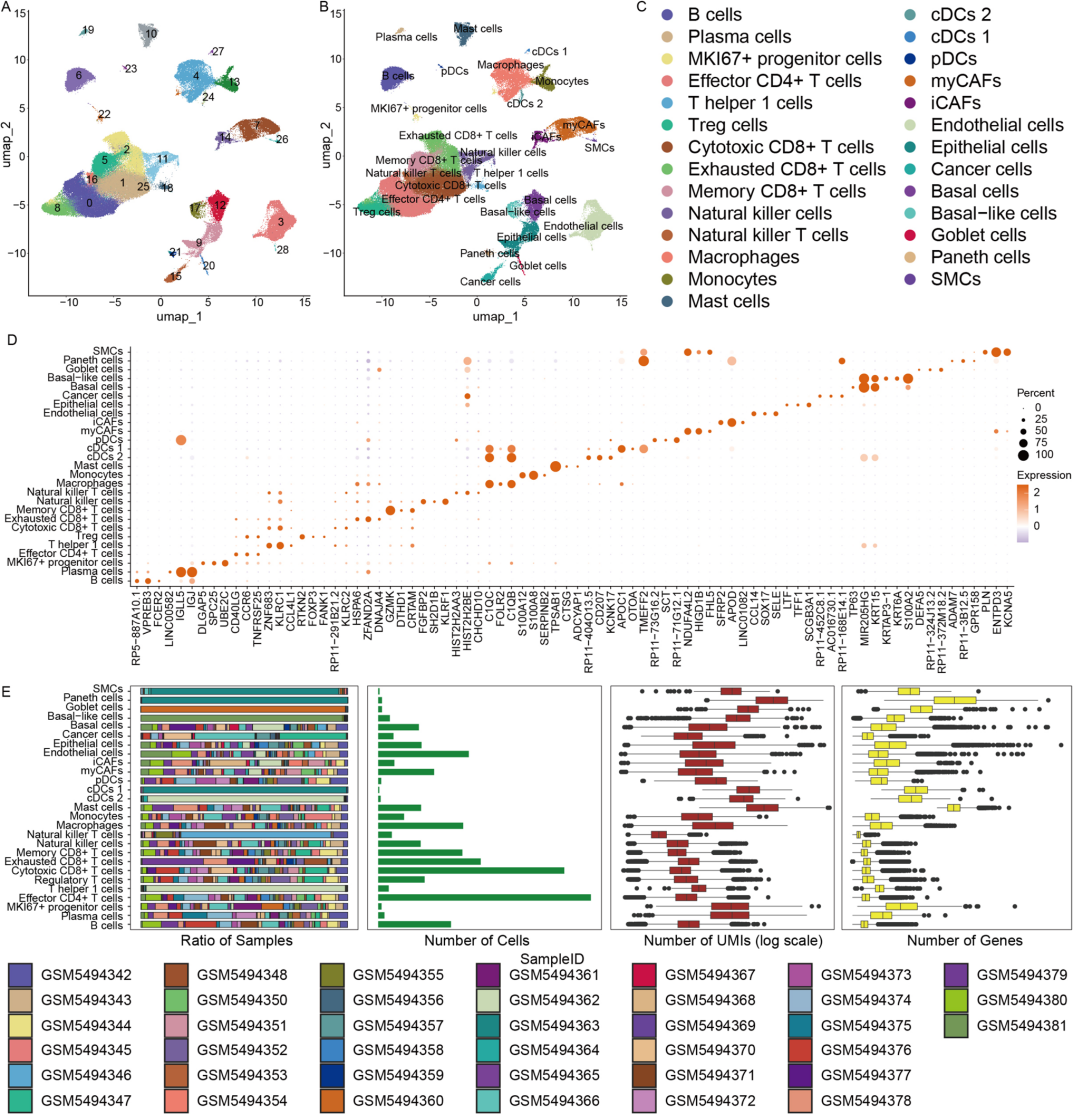
Single-cell RNA sequencing reveals the cellular heterogeneity of the TME of PC. **A** UMAP projection of all cells, illustrating 29 distinct cell clusters, with each cluster represented by a different color. **B**-**C** UMAP visualizations annotated according to major cell types. **D** Dot plot showing the differential expression of marker genes across the identified cell subsets. **E** Overview of basic characteristics of each cell subset, including (from left to right): the proportional distribution of cell subsets across individual samples (stacked bar plot); the number of cells in each subset (bar plot); the distribution of UMI counts per subset (box plot, log scale); and the distribution of detected gene numbers per subset (box plot)

Single-cell RNA sequencing data generated using the 10 × Genomics platform were obtained from a previously published study [20], comprising 39 PC samples. To minimize batch effects across samples, the single-cell datasets were integrated, ensuring that major cell-type features were comparable among different patients. After stringent quality control and filtering, a total of 171,510 immune cells were retained for unsupervised clustering analysis and were ultimately classified into 29 distinct cell clusters (Fig. 1A). Based on canonical marker genes, we successfully annotated 27 major cell subpopulations, including B cells, plasma cells, MKI67⁺ progenitor cells, effector CD4⁺ T cells, T helper 1 cells, regulatory T cells, cytotoxic T cells, exhausted T cells, memory CD4⁺ T cells, NK cells, NKT cells, macrophages, monocytes, mast cells, conventional dendritic cells type 1 (cDCs 1), conventional dendritic cells type 2 (cDCs 2), plasmacytoid dendritic cells (pDC), myofibroblastic cancer-associated fibroblasts (myCAFs), inflammatory cancer-associated fibroblasts (iCAFs), endothelial cells, epithelial cells, tumor cells, basal cells, basal-like cells, goblet cells, Paneth cells, and smooth muscle cells (Fig. 1B, C). In addition, marker genes representative of six major cell lineages was visualized (Supplementary Fig. 1A–F), including myeloid cells (CD68), B cells (CD79A), T cells (CD3E), fibroblasts (COL1A2), endothelial cells (VWF), and epithelial cells (EPCAM).

Comparative analysis revealed pronounced molecular heterogeneity among the identified cell subpopulations (Fig. 1D). Notably, cells originating from different tissues and patients exhibited substantial differences in their transcriptional characteristics (Fig. 1E). Although mast cells constituted a relatively small proportion of the total cell population, they displayed comparatively high gene expression levels, suggesting elevated cellular activity across subclusters. In contrast, effector CD4⁺ T cells, despite being one of the most abundant populations in the TME, showed lower overall transcriptional activity, indicative of a functionally suppressed or exhausted state.

### Heterogeneity of mast cells in prostate cancer

**Fig. 2:**
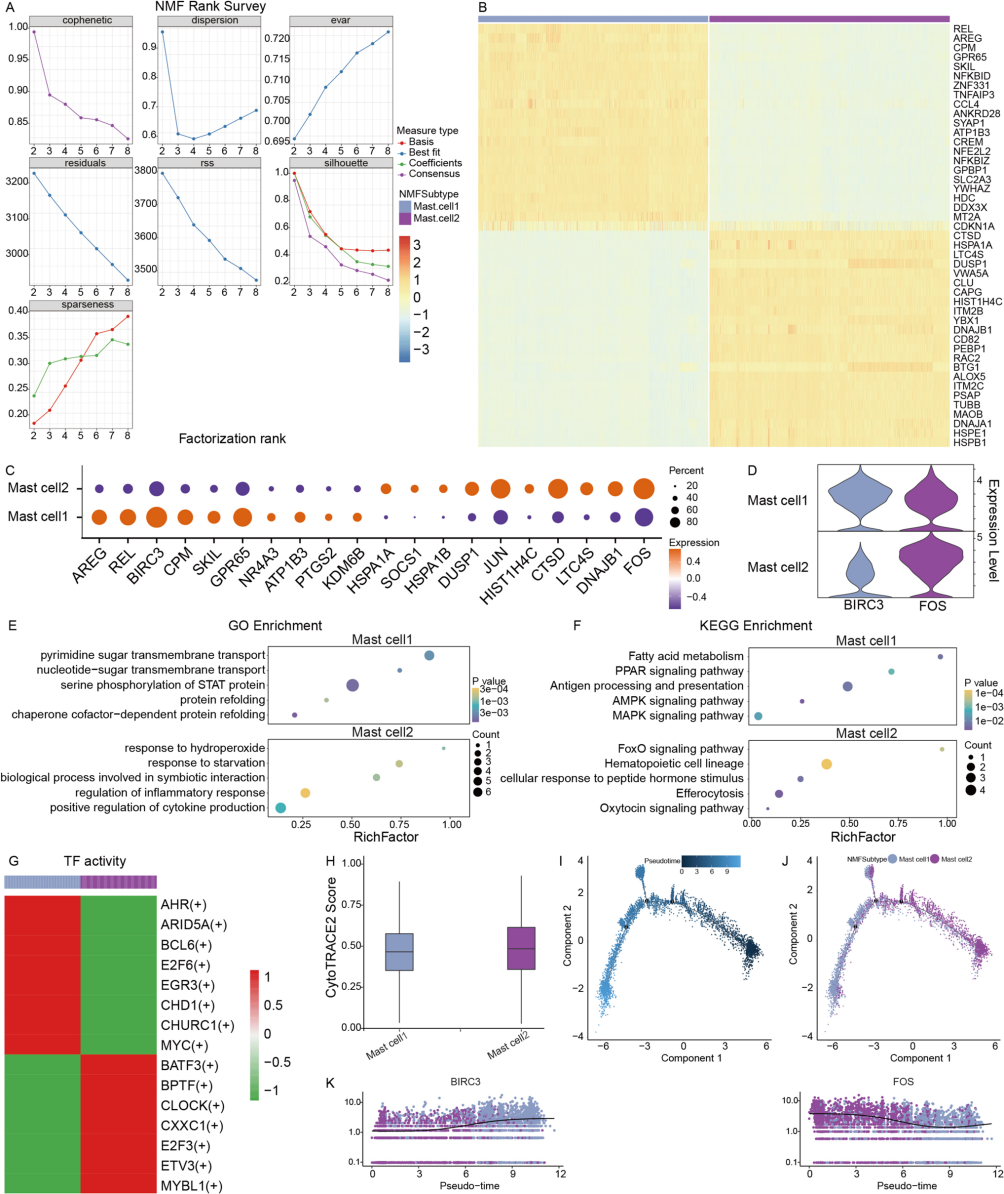
Clustering and functional characterization of mast cell subpopulations. **A** Relationship between clustering coefficients and the number of clusters (*k*) in NMF analysis of mast cells. **B** Heatmap illustrating the expression patterns of significantly differentially expressed genes between the two mast cell subsets. **C** Dot plot showing the differential expression of marker genes across the two mast cell subsets. **D** Box plots comparing the expression levels of BIRC3 and FOS between the two mast cell subsets. **E**–**F** Bubble plots depicting GO enrichment results for each mast cell subset, highlighting distinct functional annotations. **G** Heatmap of SCENIC-inferred gene regulatory networks, illustrating the activity of key TFs across the two mast cell subsets. **H** CytoTRACE2 analysis revealing differences in differentiation potential between the two mast cell subsets. **I** Monocle2-based pseudotime trajectory analysis illustrating the inferred developmental transitions among mast cell subsets, with color gradients representing pseudotime progression. **J** Annotation of mast cell subpopulations mapped onto the pseudotime trajectory. **K** Dynamic changes in the expression of mast cell subset-specific marker genes along the pseudotime axis

Given the high transcriptional activity of mast cells within the TME, we sought to investigate their functional diversity in PC by characterizing mast cell heterogeneity. A total of 6011 mast cells were subjected to clustering analysis using non-negative matrix factorization (NMF). Evaluation of clustering performance indicated that *k* = 2 was the optimal solution (Fig. 2A), resulting in the identification of two distinct mast cell subpopulations, designated mast cell1 and mast cell2. To define the molecular characteristics of each subpopulation, differential gene expression analysis was performed. Subpopulation-specific marker genes were identified based on the following criteria: avg_log2FC > 0.5, pct.1 > 0.4, and pct.2 < 0.6. As a result, 22 marker genes were identified for mast cell1 and 23 marker genes for mast cell2 (Fig. 2B). The expression patterns of the top 10 differentially expressed genes in each subpopulation were further visualized (Fig. 2C), with representative examples including BIRC3 in mast cell1 and FOS in mast cell2 (Fig. 2D).

**Fig. 3:**
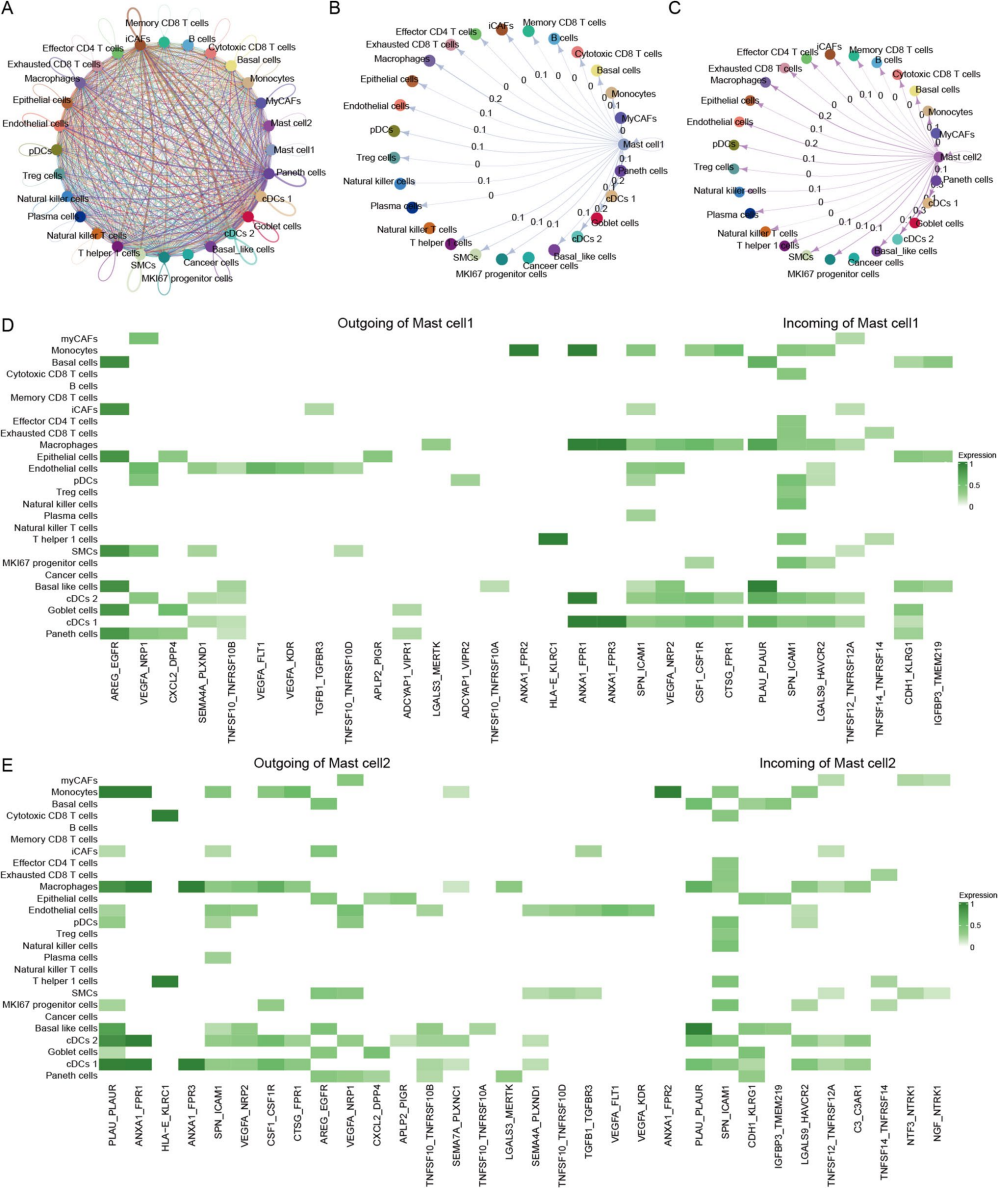
Intercellular communication features of mast cell subsets in the TME of PC. **A** Global ligand–receptor interaction network among major cell subsets within the TME of PC. **B** Cell-cell communication network centered on mast cell1, illustrating its interactions with other cell subsets. **C** Cel-cell communication network centered on mast cell2, highlighting its interaction patterns with surrounding cell populations. **D** Heatmap showing significant ligand–receptor pairs between mast cell1 and other cell subsets. Signals outgoing from mast cell1 (Outgoing of mast cell1) are displayed on the left, whereas incoming signals to mast cell1 (Incoming of mast cell1) are shown on the right. The *y-*axis represents cell types, and the *x*-axis denotes representative ligand–receptor pairs; color intensity indicates relative expression levels. **E** Heatmap illustrating significant ligand–receptor interactions between mast cell2 and other cell subsets. Signals outgoing from mast cell2 (Outgoing of mast cell2) are shown on the left, and incoming signals to mast cell2 (Incoming of mast cell2) are shown on the right, with the same color scale as in (**D**)

Subsequently, GO and KEGG pathway enrichment analyses were conducted using the subpopulation-specific marker genes, revealing marked functional divergence between the two mast cell subsets. Mast cell1 was predominantly enriched in cancer-related signaling pathways (Fig. 3E–F), including serine phosphorylation of STAT protein, the PPAR signaling pathway, and the AMPK signaling pathway. In contrast, mast cell2 showed preferential enrichment in pathways associated with inflammatory responses, such as regulation of inflammatory response and positive regulation of cytokine production, suggesting a more immunomodulatory phenotype.

Further SCENIC-based gene regulatory network analysis demonstrated distinct TF activity profiles across mast cell subsets (Fig. 2G). In mast cell1, TFs such as MYC and EGR3 exhibited high regulon specificity, implying potential roles in promoting tumor cell proliferation and growth [25, 26]. Conversely, mast cell2 displayed elevated activity of TFs including CLOCK and MYBL1. Previous studies have reported that MYBL1 can induce ANGPT2 transcription, thereby facilitating tumor angiogenesis and conferring resistance to sorafenib in hepatocellular carcinoma [27], highlighting its potential relevance in tumor progression.

To further assess the developmental status of mast cell subsets, CytoTRACE analysis was performed to estimate cellular differentiation potential. The results revealed a gradual decline in CytoTRACE scores across the two mast cell subsets, indicating that mast cell2 may represent a less differentiated state that gives rise to mast cell1 (Fig. 2H). This inferred developmental relationship was further supported by pseudotime trajectory analysis, which positioned the two subsets along a continuous differentiation axis (Fig. 2I–J). Consistently, the expression levels of subset-specific marker genes exhibited dynamic and stage-dependent changes along pseudotime (Fig. 2K).

### Mast cell subsets act as communication hubs to remodel the prostate cancer tumor microenvironment

To further elucidate intercellular communication within the tumor microenvironment (TME) of prostate cancer, we performed ligand–receptor pairing analysis to construct a cell–cell interaction network. The global network revealed extensive communication among immune cells, fibroblasts, endothelial cells, and tumor cells, within which both mast cell subsets occupied central positions and interacted with multiple cell populations (Fig. 3A). Focusing on mast cell–centered networks, we observed clear asymmetry in signaling directionality: Mast cell1 predominantly acted as a signal-sending (outgoing) population, whereas mast cell2 exhibited a stronger signal-receiving (incoming) profile (Fig. 3B–C).

To facilitate biological interpretation, we prioritized a subset of well-established and biologically relevant ligand–receptor signaling axes that distinguished the two mast cell subsets (Fig. 3D–E). Mast cell1 showed prominent outgoing interactions associated with angiogenesis, stromal remodeling, and tumor progression, including VEGFA–NRP1/FLT1 and TGFB1–TGFBR signaling, as well as cell survival and death-related pathways such as TNFSF10–TNFRSF10B/D. These signals primarily targeted tumor cells, fibroblasts, and endothelial cells, supporting a tumor-supportive communication role for mast cell1. In contrast, mast cell2 preferentially engaged in immune-related communication, particularly with T cells, NK cells, macrophages, and dendritic cells. Its outgoing interactions were enriched for inflammation-associated ligand–receptor pairs, while its incoming signals were dominated by adhesion- and survival-related cues derived from fibroblasts and tumor cells, suggesting tight regulation by the surrounding microenvironment. Together, these focused interaction patterns indicate that mast cell1 and mast cell2 represent functionally distinct communication hubs: mast cell1 primarily contributes to tumor-promoting stromal and angiogenic signaling, whereas mast cell2 is more closely associated with immune-inflammatory regulation. Through these complementary yet distinct signaling programs, the two mast cell subsets jointly participate in the dynamic remodeling of the prostate cancer TME.

### Prognostic significance of mast cell subset signatures in prostate cancer

**Fig. 4:**
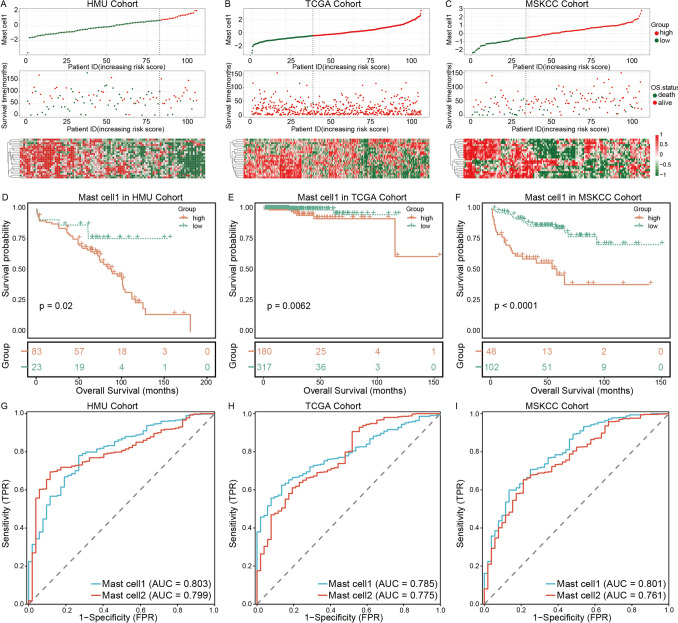
Clinical relevance of mast cell subset signatures in PC. **A**–**C**, Risk stratification based on the NESs of the mast cell1 signature in the HMU, TCGA, and MSKCC cohorts. Top panels show patients ranked by increasing risk scores, with the optimal cutoff indicated by a vertical dashed line, separating patients into low-risk (green) and high-risk (red) groups. Middle panels illustrate the relationship between risk scores and patient survival status, where green dots represent deceased patients and red dots represent surviving patients. Bottom panels display the expression patterns of marker genes across patients. **D**–**F** Kaplan-Meier survival analyses comparing overall survival between high- and low-risk groups stratified by the mast cell1 signature in the HMU, TCGA, and MSKCC cohorts. **G**–**I**, ROC curve analyses evaluating the prognostic performance of the mast cell1 and mast cell2 signatures across the HMU, TCGA, and MSKCC cohorts

To further investigate the clinical relevance of the two mast cell subsets, we collected three independent bulk RNA-seq datasets, including the HMU, TCGA, and MSKCC cohorts. Subset-specific gene signatures were constructed based on the marker genes of each mast cell subset, and single-sample gene set enrichment analysis (ssGSEA) was applied to calculate NESs for each patient. Patients were subsequently stratified into high-score and low-score groups according to the optimal cutoff value determined from their normalized enrichment scores. Figure 4A–C illustrates the distribution of enrichment scores, overall survival duration, survival status, and expression patterns of the mast cell1 signature across the three cohorts. Notably, patients in the low-score group of mast cell1 exhibited a lower mortality rate, suggesting that elevated mast cell1-associated signatures may be unfavorable for PC prognosis. In contrast, patients with higher mast cell2 enrichment scores demonstrated prolonged survival, indicating that enrichment of the mast cell2 signature may be beneficial for patient outcomes (Supplementary Fig. 2A–C). Kaplan–Meier survival analyses further supported these observations, revealing significantly shorter overall survival in patients with high mast cell1 scores across all three cohorts (Fig. 4D–F), consistent with a tumor-promoting role of the mast cell1. Conversely, patients with high mast cell2 scores showed improved overall survival, suggesting a potential tumor-suppressive effect of this subset (Supplementary Fig. 2D–F). To assess the predictive performance of mast cell subset signatures for patient prognosis, receiver operating characteristic (ROC) curve analyses were conducted. The results demonstrated that both mast cell subset signatures exhibited good prognostic discrimination, with particularly strong predictive accuracy observed in the HMU cohort (Fig. 4G–I). Importantly, multivariable Cox regression analyses incorporating established clinicopathological factors confirmed that mast cell subset scores remained independent prognostic predictors of overall survival (Supplementary Table 2).

### Expression of mast cell subset-associated genes in prostate cancer

**Fig. 5:**
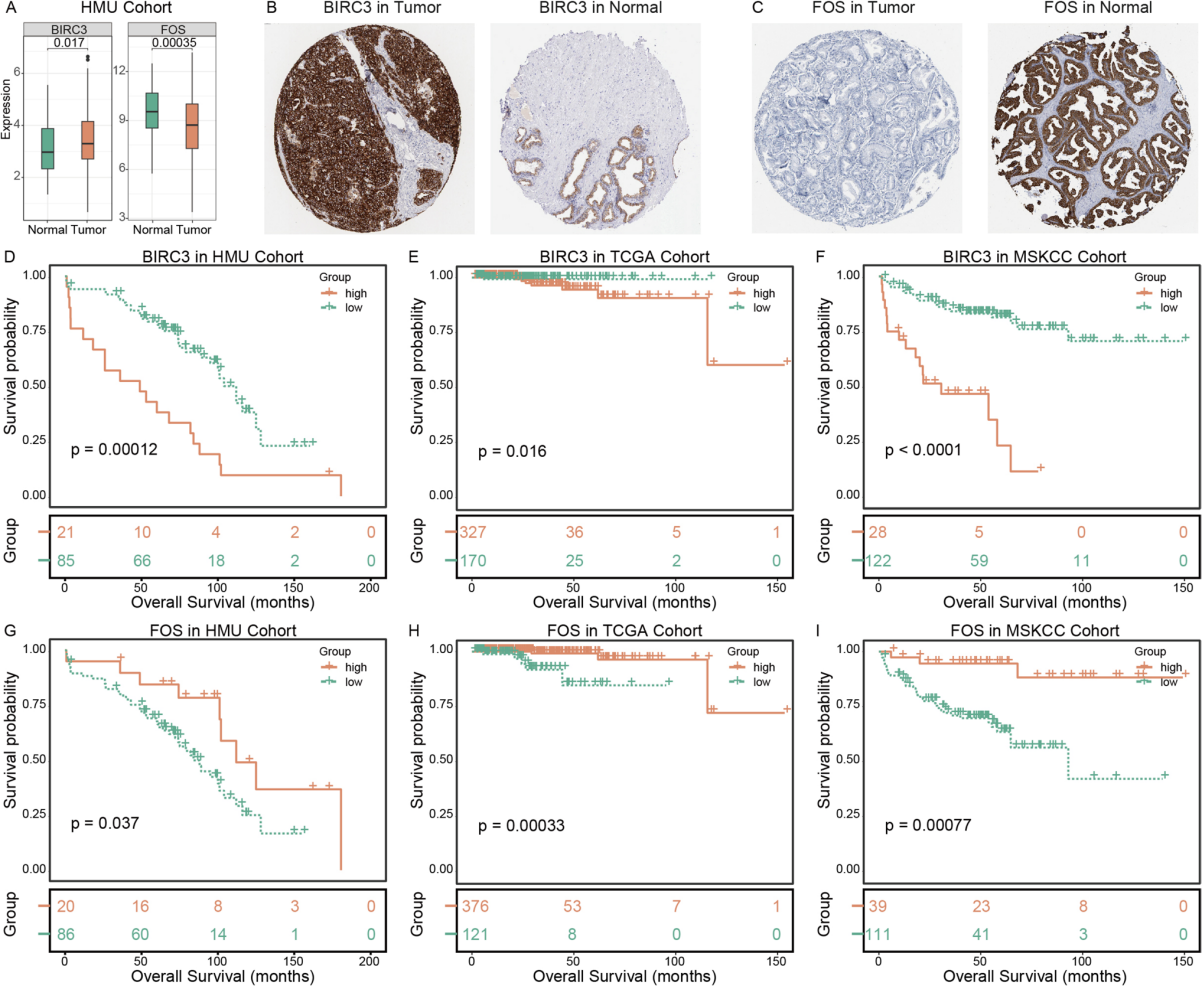
Expression patterns and prognostic significance of mast cell subset-specific marker genes. **A** Differential expression of the mast cell subset-specific marker genes BIRC3 (mast cell1) and FOS (mast cell2) between tumor and normal tissues in the HMU cohort. **B**–**C** Immunohistochemical staining showing protein expression levels of BIRC3 and FOS in PC tumor and normal tissues. **D**–**F**, Kaplan-Meier survival analyses comparing overall survival between patients with high and low BIRC3 expression in the HMU, TCGA, and MSKCC cohorts. **G**–**I**, Kaplan-Meier survival analyses comparing overall survival between patients with high and low FOS expression in the HMU, TCGA, and MSKCC cohorts

To further validate the robustness of mast cell subset-specific signatures, we compared the expression patterns of representative marker genes derived from the two mast cell subsets. The results demonstrated that BIRC3, a signature gene of the mast cell1, was significantly upregulated in tumor tissues, whereas FOS, a representative marker of the mast cell2, exhibited reduced expression in tumor samples (Fig. 5A). Consistent trends were observed at the protein level. Immunohistochemical analyses from the human protein atlas (HPA) database revealed elevated BIRC3 protein expression in PC tissues, while FOS protein expression was relatively higher in normal tissues (Fig. 5B–C).

Based on the expression levels of these genes in individual patients, optimal cutoff values were determined to stratify patients into high-expression and low-expression groups. Kaplan-Meier survival analyses showed that patients with high BIRC3 expression had significantly shorter overall survival, indicating that BIRC3 may serve as a potential risk factor in PC progression (Fig. 5D–F). In contrast, patients with high FOS expression exhibited prolonged overall survival, suggesting that FOS may function as a protective prognostic marker in PC (Fig. 5G–I). Furthermore, multivariable Cox regression analyses demonstrated that both BIRC3 and FOS expression levels were independent prognostic factors after adjustment for conventional clinical variables (Supplementary Table 3).

## Discussion

In this study, we integrated single-cell and bulk transcriptomic data to comprehensively characterize the heterogeneity of mast cell subsets within the TME of PC and to elucidate their distinct prognostic implications. Our findings revealed a complex and context-dependent functional landscape of mast cells in PC, underscoring their multifaceted roles in tumor progression and patient outcomes.

Mast cells are a critical immune component of the TME, yet their role in PC has long been controversial. Previous studies have suggested that mast cells may exert dual effects in cancer biology. On the one hand, mast cells can promote tumor progression by releasing histamine, proteases, chemokines, and other pro-tumorigenic mediators that facilitate angiogenesis, invasion, and metastasis. On the other hand, accumulating evidence indicates that mast cells may also suppress tumor growth by inducing endoplasmic reticulum stress or directly promoting tumor cell apoptosis [28]. For instance, recent work has demonstrated that mast cell-derived Cystatin C significantly inhibits PC progression through the induction of endoplasmic reticulum stress, highlighting a potential antitumor role of mast cells [29]. Consistent with these observations, our study revealed pronounced functional heterogeneity among mast cell subsets. Specifically, the mast cell1 was strongly associated with tumor-promoting signaling pathways, including STAT, MAPK, and FoxO pathways, whereas the mast cell2 was predominantly involved in immune and inflammatory regulation and antitumor immune responses. These findings suggest that mast cells may concurrently exert pro-tumorigenic and antitumor effects in PC, reflecting their functional plasticity and contextual dependency within the TME.

Compared with previously published single-cell studies, our work further extends the understanding of mast cell heterogeneity at the transcriptomic level. A prior single-cell study by Xie et al. reported that mast cells in colorectal cancer exhibit activated phenotypes characterized by high expression of TPSAB1, CPA3, and KIT and are associated with favorable prognosis [28]. In line with these findings, we observed activation of mast cell subset-specific gene expression in PC. Importantly, our study goes beyond marker identification by delineating functional divergence and developmental trajectories between mast cell subsets. Our analyses indicate that the mast cell2 represents a less differentiated state that progressively transitions toward the mast cell1. Moreover, through SCENIC-based gene regulatory network analysis, we systematically identified subset-specific transcriptional regulators, revealing distinct regulatory activities of key TFs such as MYC, EGR3, CLOCK, and MYBL1 across mast cell subsets. These findings provide mechanistic insights into mast cell functional diversification and highlight potential therapeutic targets for precision therapy in PC.

Most previous studies assessing mast cells in PC relied primarily on immunohistochemical quantification of mast cell density and its association with clinical outcomes [30]. However, these studies have yielded inconsistent conclusions, with some reporting a favorable prognostic impact of high mast cell infiltration, while others associating it with poor prognosis. Such discrepancies may arise from methodological differences, cohort heterogeneity, and spatial variability of mast cell localization within tumor tissues (e.g., intratumoral versus peritumoral regions). By integrating single-cell and bulk transcriptomic analyses, our study overcomes limitations related to spatial localization and provides a refined molecular framework linking mast cell subset-specific gene signatures to patient prognosis. Importantly, our findings clarify the differential prognostic contributions of distinct mast cell subsets, offering a novel perspective to reconcile conflicting results from previous studies.

In this study, we combined CytoTRACE and pseudotime trajectory analyses to explore the transcriptional relationships among mast cell subsets in the tumor microenvironment of prostate cancer. While these analyses suggested an ordered relationship between mast cell2 and mast cell1, this finding should be interpreted with caution. Mast cells are generally considered terminally differentiated immune cells; therefore, the application of developmental inference tools such as CytoTRACE and Monocle in this context does not necessarily imply a classical lineage differentiation process. Rather than reflecting true developmental hierarchies, the inferred trajectories are more likely to capture continuous transcriptional and functional state transitions within mature mast cell populations. In this framework, the observed gradients in CytoTRACE scores and the dynamic changes of subset-specific marker genes along pseudotime may represent differences in activation status, functional polarization, or microenvironment-driven remodeling of mast cell functions. This perspective extends previous studies that largely treated mast cells as a homogeneous population, highlighting the potential functional plasticity of mast cells within the prostate cancer microenvironment. Taken together, our analyses suggest that mast cell functions may dynamically evolve along transcriptional state continua rather than strict differentiation trajectories. Future studies integrating spatial analyses, lineage-tracing strategies, or experimental validation will be required to further clarify the biological nature and clinical relevance of these mast cell states.

Nevertheless, several limitations of this study should be acknowledged. First, the sample size of our cohorts was relatively limited and primarily derived from a single medical center, which may restrict the generalizability of our findings. Validation in larger, multi-center, independent cohorts is therefore warranted. Second, while our transcriptomic analyses provide robust associations, many of the conclusions, particularly those regarding subset-specific marker genes, TFs, and prognostically relevant genes such as BIRC3 and FOS, require further experimental validation to elucidate their precise functional roles and mechanistic contributions. In particular, the lack of in vitro or coculture-based functional assays limits causal inference regarding the roles of these genes in mast cell-mediated tumor regulation. Third, the identification of mast cell1 and mast cell2 was based on transcriptomic clustering without direct in situ validation. Although complementary bulk RNA-seq analyses showed consistent associations between subset-specific marker expression and corresponding mast cell subset scores, future spatial and experimental studies will be necessary to fully validate the biological and clinical relevance of these mast cell subpopulations.

## Conclusion

We identified two distinct mast cell subsets with opposing functional characteristics, mast cell1, which exhibits pronounced tumor-promoting features, and mast cell2, which predominantly displays antitumor immunoregulatory properties. Elevated expression of the mast cell1 signature was strongly associated with poorer overall survival, whereas higher enrichment of the mast cell2 signature correlated with more favorable clinical outcomes. Moreover, differential expression of mast cell subtype-specific marker genes further substantiated these findings. Specifically, high BIRC3 expression and low FOS expression were significantly associated with adverse prognosis, reinforcing their potential roles as key mediators of mast cell-driven tumor progression. Collectively, these results provide novel molecular insights into mast cell heterogeneity in PC and identify promising candidate biomarkers and therapeutic targets for clinical diagnosis, prognostic stratification, and the development of personalized treatment strategies.

## Supplementary Information

Below is the link to the electronic supplementary material.

Supplementary file1 (JPG 695 kb)

Supplementary file2 (JPG 1457 kb)

Supplementary file3 (XLSX 12 kb)

Supplementary file4 (XLSX 11 kb)

Supplementary file5 (XLSX 9 kb)

## Data Availability

The human sequence data generated in this study are not publicly available due to patient privacy requirements, but are available upon reasonable request from the corresponding author. Other data generated in this study are available from GEO, cBioportal and TCGA.
