# Supplementary figures and images for: Heterogeneity and prognostic significance of mast cell subsets in the tumor microenvironment of prostate cancer
Source: Cancer Immunol Immunother. 2026 Mar 24;75(4):118. doi: 10.1007/s00262-026-04363-6 (PMC13013976; doi:10.1007/s00262-026-04363-6)

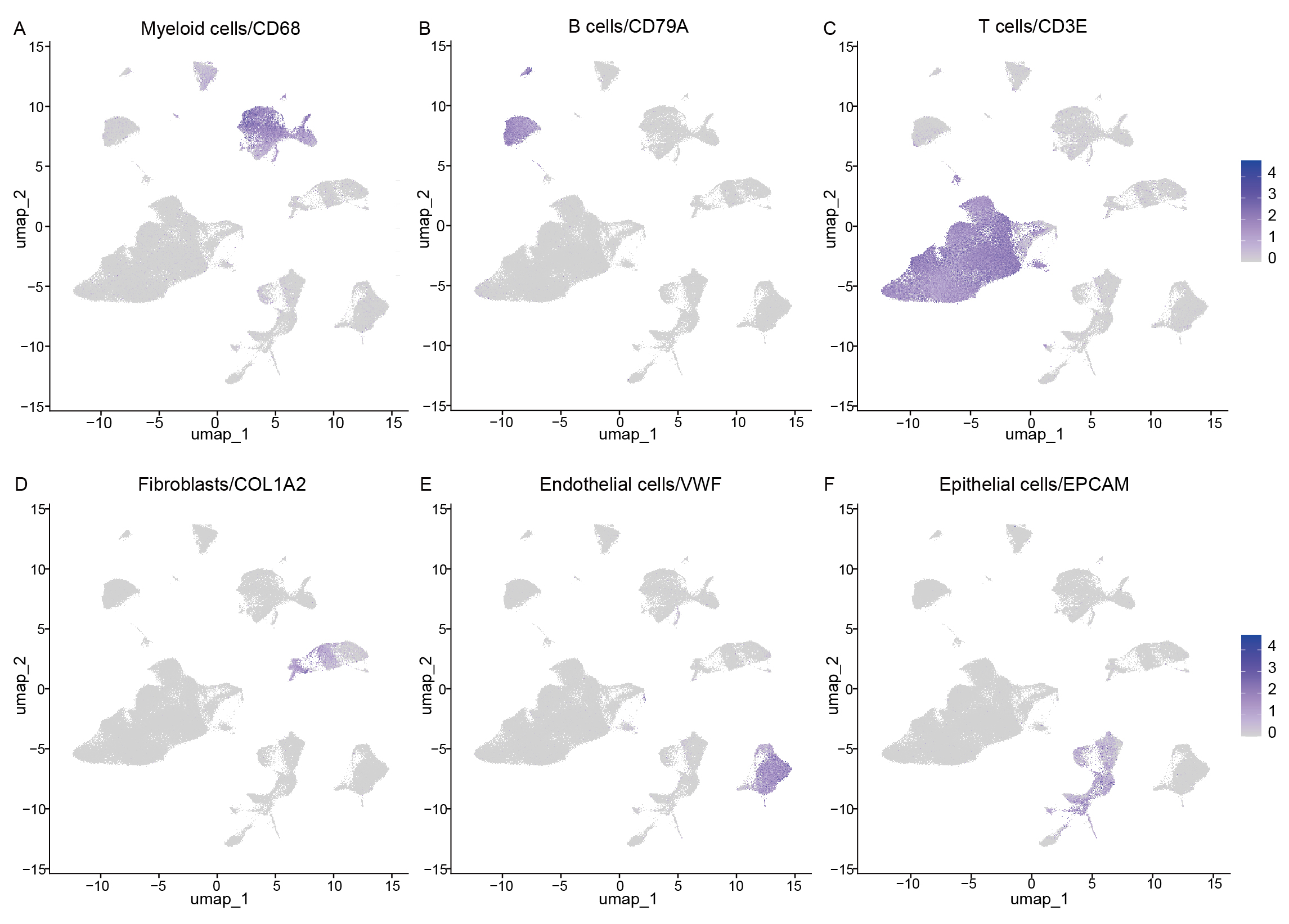

Supplement: Supplementary file 1 — Supplementary file1 (JPG 695 kb) [file 262_2026_4363_MOESM1_ESM.jpg]

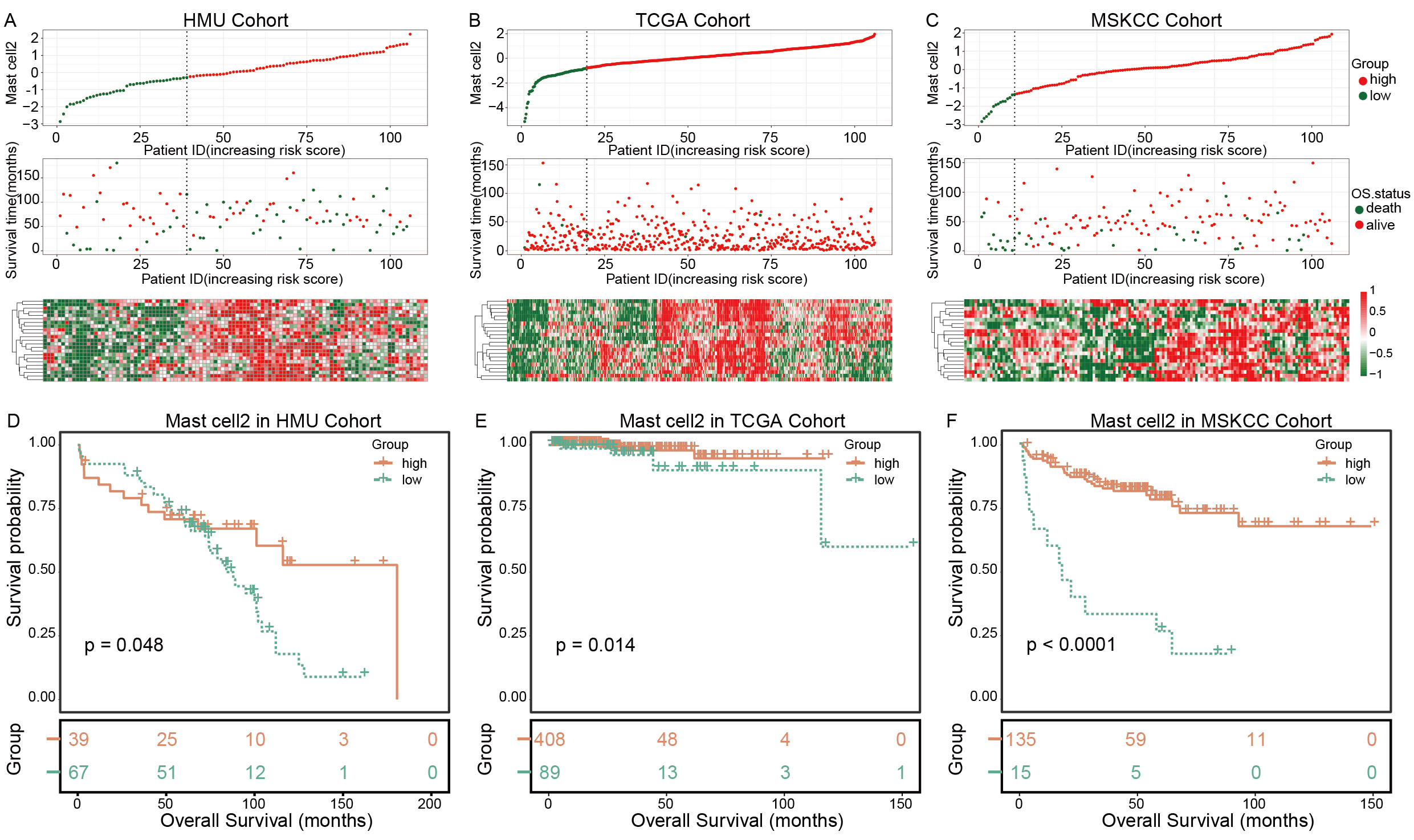

Supplement: Supplementary file 2 — Supplementary file2 (JPG 1457 kb) [file 262_2026_4363_MOESM2_ESM.jpg]
